# Lack of association between *Toxoplasma gondii* exposure and depression in pregnant women: a case-control study

**DOI:** 10.1186/s12879-017-2292-1

**Published:** 2017-03-06

**Authors:** Cosme Alvarado-Esquivel, Ana Liliana Martínez-Martínez, Luis Francisco Sánchez-Anguiano, Jesús Hernández-Tinoco, Juan Manuel Castillo-Orona, Carlos Salas-Martínez, Antonio Sifuentes-Álvarez, Ada Agustina Sandoval-Carrillo, José M. Salas-Pacheco, Oliver Liesenfeld, Elizabeth Irasema Antuna-Salcido

**Affiliations:** 1 Faculty of Medicine and Nutrition, Juárez University of Durango State, Avenida Universidad S/N, 34000 Durango, Dgo Mexico; 2 Institute for Scientific Research “Dr. Roberto Rivera Damm”, Juárez University of Durango State, Avenida Universidad S/N, 34000 Durango, Durango Mexico; 3 General Hospital, Secretary of Health, Avenida 5 de febrero 220, 34000 Durango, Mexico; 4 Institute for Microbiology and Hygiene, Campus Benjamin Franklin, Charité Medical School, Hindenburgdamm 27, D-12203 Berlin, Germany; 5 Laboratorio de Investigación Biomédica, Facultad de Medicina y Nutrición, Avenida Universidad S/N, 34000 Durango, Dgo Mexico; 6 Present Address: Medical and Scientific Affairs, Roche Molecular Systems, Pleasanton, CA 94588 USA

**Keywords:** *Toxoplasma gondii*, Seroprevalence, Depression, Pregnant women, Case-control study

## Abstract

**Background:**

Very little is known about the link of *T. gondii* infection and depression. Through an age-, gender-, and month of pregnancy-matched case-control study, we determined the association of *T. gondii* infection and depression in pregnant women.

**Methods:**

We studied 200 pregnant women with depression and 200 pregnant women without depression attended in a public hospital in Durango City, Mexico. Pregnant women were tested for the presence of anti-*Toxoplasma* IgG antibodies using an enzyme-linked immunoassay (EIA), and IgG seropositive women were further tested for the presence of IgM using an EIA. IgM positivity by EIA was further analyzed by enzyme-linked fluorescence assay (ELFA).

**Results:**

Anti-*T. gondii* IgG antibodies were found in 9 (4.5%) of the 200 cases and in 12 (6.0%) of the 200 controls (OR = 0.73; 95% CI: 0.30–1.79; *P* = 0.50). The frequency of high (>150 IU/ml) anti-*T. gondii* IgG levels was similar in cases and in controls (OR = 1.20; 95% CI: 0.36–4.01; *P* = 0.75). Two women were positive for IgM by EIA but both were negative by ELFA.

**Conclusions:**

We did not find serological evidence of an association between *T. gondii* infection and depression in pregnant women attended in a public hospital in Durango City, Mexico. Since an association of *T. gondii* and depression in pregnancy has been reported in the U.S. previously, further research to elucidate the role of *T. gondii* in prenatal depression should be conducted.

**Electronic supplementary material:**

The online version of this article (doi:10.1186/s12879-017-2292-1) contains supplementary material, which is available to authorized users.

## Background

*Toxoplasma gondii* (*T. gondii*) is a widely-distributed parasite [1], transmitted to humans by ingestion of raw or undercooked meat containing tissue cysts, and ingestion of food or water contaminated with oocysts shed by cats [2, 3]. Primary infections in pregnant women may result in vertical transmission leading to congenital infections and disease [4, 5]. Although most infections with *T. gondii* are asymptomatic, some infected individuals develop a disease called toxoplasmosis with involvement of eyes, lymph nodes and central nervous system [6, 7]. Immunocompromised individuals infected with *T. gondii* are at risk for a reactivation of the infection leading to a severe disease mainly of the central nervous system [8]. Infection with *T. gondii* has been linked to psychiatric disorders including schizophrenia [9, 10], bipolar disorder, obsessive-compulsive disorder, and addiction [9]. However, the link between *T. gondii* infection and depression is controversial. In a Cuban study of psychiatric patients, those suffering from depressive disorders had the highest frequency of reactivity to the toxoplasmin intradermal test [11]. However, in a population-representative birth-cohort of individuals in Dunedin, New Zealand, *T. gondii* seropositivity was not significantly associated with major depression [12]. Similarly, in a meta-analysis of 50 studies into *T. gondii* infection for major psychiatric disorders versus healthy controls, no association between *T. gondii* IgG seroprevalence and major depression was found [9]. The association of *T. gondii* infection and depression in pregnant women has been poorly studied. Groër et al. found that higher anti-*T. gondii* IgG titers in infected women in the USA were related to depression and anxiety during pregnancy [13]. We aimed to determine whether *T. gondii* infection is associated with depression in pregnant women attended in a public hospital in Durango City, Mexico.

## Methods

### Study design and population studied

We performed an age-, gender-, and month of pregnancy-matched case-control study of 200 pregnant women suffering from depression and 200 pregnant women without depression attended in a public hospital in Durango City, Mexico. This study was performed from March 2015 to February 2016. Inclusion criteria for enrollment of participants were: 1) pregnant women suffering from depression attending prenatal care consultations in the General Hospital of the Secretary of Health in Durango City; 2) aged 13 years and older; and 3) who accepted to participate in the study. Socioeconomic status was not a restrictive criterion for enrollment. Mean age in cases was 23.40 ± 8.36 (range 13–43) years old. Depressed pregnant women had 2–8 months of pregnancy (mean 6.5 ± 1.5 months). As a strategy to screen depression in pregnant women, validated Mexican versions of the Edinburgh postnatal depression scales (EPDS) (Additional file 1) were used in adults [14] and teenagers [15]. Pregnant women who screened positive for depression in the EPDS were further examined by a psychiatrist to confirm depression using the Diagnostic and Statistical Manual of Mental Disorders, Fifth edition criteria [http://www.dsm5.org/Pages/Default.aspx]. Control pregnant women were matched with cases for age. Controls were randomly selected, and they scored negative for depression in the EPDS. Inclusion criteria for enrollment of control pregnant women were: 1) pregnant women without depression attending prenatal consultations in the General Hospital of the Secretary of Health in Durango City; 2) aged 13 years and older; and 3) who accepted to participate in the study. Mean age in control women was 23.01 ± 7.55 (range 13–45) years old. Pregnant women without depression had 2–9 months of pregnancy (mean 6.7 ± 1.5 months). No statistically significant differences in age (*P* = 0.62), and month of pregnancy between cases and controls were found.

### Detection of anti-*T. gondii* antibodies

Serum samples from participants were obtained and stored at −20 °C until analyzed. The presence of anti-*T. gondii* IgG antibodies was tested in sera using the commercially available enzyme immunoassay (EIA) kit “*Toxoplasma* IgG” (Diagnostic Automation/Cortez Diagnostics Inc., Woodland Hills, CA, USA). Anti-*T. gondii* IgG antibody levels were expressed as International Units (IU)/ml, and a cut-off for seropositivity of 8 IU/ml was used. Sera positive for anti-*T. gondii* IgG antibodies were further tested for anti-*T. gondii* IgM antibodies by using the commercially available EIA “*Toxoplasma* IgM” kit (Diagnostic Automation/Cortez Diagnostics Inc.). In addition, sera positive for anti- *T. gondii* IgM antibodies by EIA were further analyzed for these anti-*T. gondii* IgM antibodies using the commercially available enzyme-linked fluorescent assay (ELFA) kit “VIDAS Toxo IgM” (BioMérieux, Marcy-l’Etoile, France). All IgG and IgM assays were performed following the instructions of the manufacturers.

### Statistical analysis

Analysis was conducted using the software Epi Info 7 and SPSS 15.0 (SPSS Inc. Chicago, Illinois). For calculation of the sample size, we used a 95% confidence level, a power of 80%, a 1:1 proportion of cases and controls, a reference seroprevalence of 6.1% [16] as the expected frequency of exposure in controls, and an odds ratio of 2.8. The result of the sample size calculation was 195 cases and 195 controls. We used the student’s *t* test to compare the age among cases and controls. The association of *T. gondii* infection and depression in pregnant women was assessed with the two-tailed Pearson’s chi-squared test. Odds ratio (OR) and 95% confidence interval (CI) were calculated, and a *P* value < 0.05 was considered statistically significant.

## Results

Of the 200 cases of depression included in the study, 122 (61.0%) suffered from minor depression, and 78 (39.0%) from major depression. Anti-*T. gondii* IgG antibodies were found in 9 (4.5%) of the 200 cases and in 12 (6.0%) of the 200 controls. The seroprevalence of *T. gondii* infection was similar in cases and in controls (OR = 0.73; 95% CI: 0.30–1.79; *P* = 0.50). Of the 9 anti-*T. gondii* IgG positive cases, 6 (66.7%) had IgG levels higher than 150 IU/ml, one (11.1%) between 100 and 150 IU/ml, and 2 (22.2%) between 8 and 99 IU/ml. In contrast, of the 12 anti-*T. gondii* IgG positive controls, 5 (41.7%) had IgG levels higher than 150 IU/ml, one (8.3%) between 100 and 150 IU/ml, and 6 (50.0%) between 8 and 99 IU/ml. The frequency of high (>150 IU/ml) anti-*T. gondii* IgG levels was similar in cases and in controls (OR = 1.20; 95% CI: 0.36–4.01; *P* = 0.75). Seroprevalence of *T. gondii* infection in patients with minor depression (4/122: 3.3%) was comparable to that (5/78: 6.4%) found in patients with major depression (*P* = 0.29). Stratification by age groups (13–30 years, and older than 30 years) did not show differences (*P* > 0.05) in seroprevalences among cases and controls (3/149: 2.0% versus 10/159: 6.3%, and 6/51: 11.8% versus 2/41: 4.9%, respectively). Stratification by month of pregnancy groups (2–5 months, and more than 5 months) did not show differences (*P* > 0.05) in seroprevalences (2% versus 8.7%, and 5.4% versus 5.2%, respectively) or high IgG antibody levels (2% versus 4.3%, and 3.4% versus 1.9%, respectively) among cases and controls. None of the 9 anti-*T. gondii* IgG seropositive cases was reactive to anti-*T. gondii* IgM antibodies by EIA. Whereas 2 of the 12 anti-*T. gondii* IgG seropositive controls were reactive to IgM by EIA. These 2 IgM positive sera by EIA were negative to IgM by ELFA. Thus, none of the cases and controls was considered seropositive to IgM.

## Discussion

Studies about the association of *T. gondii* infection and depression have shown conflicting results [9, 11, 12]. In addition, the association of *T. gondii* infection and prenatal depression has been poorly studied in particular. Therefore, the present study aimed to determine whether *T. gondii* infection is associated with depression in a sample of pregnant women in Durango City, Mexico. Results of tests for detection of *T. gondii* performed in the present study included qualitative and quantitative IgG and IgM assays. Our results do not point towards an increased rate of depression in pregnant women infected with *T. gondii* compared to matched control patients attended in the same hospital. We are aware of only one study on the link of *T. gondii* infection and depression in pregnancy. In such study, 414 women at 16–25 weeks of gestation in the USA were examined, and researchers found that higher *T. gondii* IgG antibody titers were associated with prenatal depression [13]. Authors hypothesized that immune escape of *T. gondii* may occur due to immune changes in pregnancy, and this could cause depression through activation of indoleamine 2, 3-dehydroxylase resulting in serotonin decrease [13]. It is not clear why the association of infection and depression was found in pregnant women in the USA but not in pregnant women in the current study. Comparison of both studies was based on IgG seropositivity; however, IgM seropositivity was not compared because this marker for acute or recent infection was not determined in the American study. It is possible that differences in the characteristics of pregnant women among the studies may explain the differences in the association. In the U.S. study, researchers examined women at 16–25 weeks of pregnancy whereas we examined women at 2–9 months of pregnancy. Stratification by month of pregnancy groups (2–5 months, and >5 months) did not show an association of infection and prenatal depression in the current study. While our study did not find any association of infection with *T. gondii* and depression in pregnant women several studies have demonstrated a link between *T. gondii* infection and depression. *T. gondii* seropositivity correlated with the Center for Epidemiologic Studies Depression score, Profile for Mood States-depression, and total mood disturbance score in a study of women veterans in the USA [17]. The age of women in the latter study was higher than in our study. Experimental evidence exists that reactivation of chronic *T. gondii* infection in mice by an immunosuppressive regimen caused depression-like behaviors, specifically, reduced sucrose preference, and increased immobility in the forced-swim test [18]. Researchers of the latter study also observed an enhanced tryptophan catabolic shunt and serotonin turnover that may be involved in the development of the depressive-like behaviors [18]. Reactivation of latent infection in humans is often observed in immunocompromised patients leading to life-threatening toxoplasmic encephalitis; it is therefore difficult to study an association with depression.

False positive results have been reported in anti-*T. gondii* IgM antibody tests [19]. Therefore, to increase the specificity of IgM seropositivity, we used two methods to test for anti-*T. gondii* IgM antibodies (EIA and ELFA). No acute cases of *T. gondii* infection were found, and therefore, treatment against *T. gondii* in the pregnant women studied was not needed.

Our study has limitations. First, we investigated the association of infection with *T. gondii* and depression in a relatively small cohort of pregnant women attending a public hospital. Therefore, our results cannot be extrapolated to pregnant women with different social status, i.e., those attended in private hospitals or other public hospitals. The great majority of women attended in the participating hospital had a low socioeconomic status.

## Conclusions

We did not observe serological evidence of an association between *T. gondii* infection and depression in pregnant women attended in a public hospital in Durango City, Mexico. Our results conflict with those reported in a previous study in the USA therefore warranting further research to elucidate the role of *T. gondii* in prenatal depression.

## Additional file

Additional file 1: Tool used to screen depression. (DOCX 15 kb)

## Abbreviations

CI: Confidence interval
EIA: Enzyme-linked immunoassay
ELFA: Enzyme-linked fluorescence assay
EPDS: Edinburg postnatal depression scale
IU: International units
ml: Milliliter
OR: Odds ratio
SPSS: Statistical package for the Social Sciences
USA: United States of America
