# Supplementary material for: Lack of association between Toxoplasma gondii exposure and depression in pregnant women: a case-control study
Source: BMC Infect Dis. 2017 Mar 6;17:190. doi: 10.1186/s12879-017-2292-1 (PMC5340021; doi:10.1186/s12879-017-2292-1)
Supplement: Additional file 1: — Tool used to screen depression. (DOCX 15 kb) [file 12879_2017_2292_MOESM1_ESM.docx]

Nombre: No. de folio:

Nos gustaría saber cómo se ha estado sintiendo. Por favor **subraye** la respuesta que más se acerca a como se ha sentido **en los últimos 7 días**.

1. He podido reír y ver el lado bueno de las cosas:

Tanto como siempre 0

No tanto ahora 1

Mucho menos 2

No, no he podido. 3

2. He mirado el futuro con placer:

Tanto como siempre 0

Algo menos que antes 1

Definitivamente menos que antes 2

No, nada 3

3. Me he culpado innecesariamente cuando las cosas marchaban mal:

Sí, la mayoría de las veces 3

Si, algunas veces 2

No muy frecuentemente 1

No, nunca 0

4. He estado ansiosa y preocupada sin motivo:

No, nada 0

Rara vez 1

Sí, a veces 2

Sí, muy frecuentemente 3

5. He sentido miedo o pánico sin motivo alguno:

Sí, muy frecuentemente 3

Sí, a veces 2

No, no mucho 1

No, nada 0

6. Las cosas me han estado agobiando:

Sí, casi siempre 3

Sí, a veces 2

No, casi nunca 1

No, nada 0

7. Me he sentido tan infeliz, que he tenido dificultad para dormir:

Sí, casi siempre 3

Sí, a veces 2

No muy frecuentemente 1

No, nada 0

8. Me he sentido triste o miserable:

Sí, casi siempre 3

Sí, muy frecuentemente 2

No muy frecuentemente 1

No, nada 0

9. He estado tan infeliz que he estado llorando:

Sí, casi siempre 3

Sí, muy frecuentemente 2

Sólo ocasionalmente 1

No, nunca 0

10. He pensado en hacerme daño a mi misma:

Sí, muy frecuentemente 3

A veces 2

Rara vez 1

Nunca 0
